# Behavior and anxiety levels in pediatric patient: The behavioral changes and anxiety of pediatric patient in dental clinic

**DOI:** 10.1002/cre2.795

**Published:** 2023-10-15

**Authors:** Rana A. Al Homoud, Amani K. Alshellatie, Abdullah S. Alzumaie, Sura A. Al‐Bayati

**Affiliations:** ^1^ Gulf Medical University, DMD Saihat Saudi Arabia; ^2^ Gulf Medical University, DMD Safwa Saudi Arabia; ^3^ Gulf Medical University, BDS Ajman United Arab Emirates; ^4^ Diagnostic and Surgical Dental Science Department College of Dentistry, Gulf Medical University, BDS Ajman United Arab Emirates

**Keywords:** anxiety, behavior rating scales, child behavior, child behavior control

## Abstract

**Aim:**

The aim of this study is to evaluate the behavioral patterns and anxiety levels of pediatric patients in dental clinics, discern their behavioral expectations, and investigate the associations between these factors and the patient's age and gender.

**Methods:**

In this cross‐sectional study, 150 pediatric patients visiting Sharjah Thumbay Dental Hospital for treatment were recruited over a study period of 23 weeks (February 12, 2022 to July 23, 2022). The patient's age ranged from 2 to 14 years. Guardians were informed about the study and their written consent was taken.

**Exclusion Criteria:**

Children over 14 years old, children whose guardians refused to participate in the study, and medically compromised children. Frankl's behavior rating scale, Categorical rating scale, and Venham anxiety and behavioral rating scales were used to evaluate the pediatric patient's behavior and anxiety at the end of the dental visit, the evaluation was done by the same trained dental student for all the patients.

**Results:**

When the Mann–Whitney *U* test was used for gender, no significant differences were observed in behavior and anxiety between male and female patients across all scales employed in this study. Conversely, when examining various age groups for behavior and anxiety using the Kruskal–Wallis test, significant findings emerged across nearly all scales. In the categorical rating scale, the age group of 11–14 years exhibited the most notable results in subscales of crying (*p* = .034), cooperativeness (*p* = .002), and apprehensiveness (*p* = .003).

**Conclusion:**

The pediatric patients who took part in this study exhibited heightened anxiety when attending dental clinics. This study underscores the importance of understanding child behavior and utilizing effective communication with children and their guardians. Dental professionals should consider implementing strategies to manage child behavior during visits. Further research is required to develop sufficient strategies tailored to different pediatric populations, aiming to enhance dental care outcomes for pediatric patients.

## 1 BACKGROUND

In the sphere of pediatric dentistry, a great deal of emphasis is placed not only on conducting dental procedures but also on providing effective guidance to pediatric patients during their dental visits. The goal is to create positive experiences and foster a favorable attitude toward dental health. Evaluating dental anxiety and monitoring behavioral changes in pediatric patients are of paramount importance, holding equal significance to the treatment itself. This is because a thorough understanding of a child's anxiety levels and behavioral patterns can boost the confidence of dental professionals. Moreover, it allows for the development of bespoke treatment management strategies, specifically designed for each individual pediatric dental patient (Shetty et al., 2015).

A vital skill for dentists is assessing the behavior of each child. Dental anxiety and behavioral changes during treatment are considered significant challenges for successful patient management and treatment completion (Al‐Namankany et al., 2012). Various behavior evaluation scales, such as Frankl's behavior rating scale (FBRS), Categorical rating scale, and Venham anxiety and behavioral rating scale (described below), have been developed to analyze pediatric patients' conduct during dental appointments, assisting dentists in providing appropriate treatment. Factors like child age, parental behavior, and parental anxiety can impact a child's behavior in the dental clinic (Tyagi & Sharma, 2011).

### 1.1 Frankl's behaviour rating scale (FBRS)

Introduced by Frankl in 1962, the FBRS is among the most utilized behavioral evaluation scales in both research and routine clinical practice. This widely utilized behavioral evaluation scale, both in research and routine clinical practice, categorizes a child's behavior into four groups according to their demeanor during dental procedures. It comprises four behavior categories, spanning from strongly positive to strongly negative, which are assessed by the treating clinician and can be employed at various stages of therapy. The FBRS is recognized as one of the most effective tools for evaluating children's behavior in dental clinics (Narayan & Samuel, 2019).

### 1.2 Categorical rating scale

Developed by Nazif in 1971, the categorical rating scale is widely employed by researchers. This scale analyzes and records behavior during dental visits using four elements: crying, compliance, apprehensiveness, and sleepiness. The results of the four scale elements are combined to yield a total time point value. Moreover, this scale is applied to assess a patient's response to specific procedures, such as the administration of local anesthetic medication. The categorical rating scale has been demonstrated to be a valid instrument for evaluating patient behavior (Narayan & Samuel, 2019).

### 1.3 Venham anxiety and behavioral rating scale

This scale consists of two subscales: Venham Anxiety and Venham Behavioral. Both subscales evaluate a child's fear and lack of cooperation within the clinical setting. Each measure contains five behaviorally defined categories ranging from zero to five, with higher values indicating increased anxiety or uncooperative demeanor. These scales have been shown to provide accurate evaluations, even when used by novice clinicians (Narayan & Samuel, 2019).

Despite the development of numerous approaches for managing pediatric dental patients, including nonpharmacological techniques and behavioral modeling methods, it remains unclear which factors most substantially influence children's behavior during dental appointments and which strategies are most effective for managing dental anxiety and behavior across different pediatric populations.

Previous dental experiences have been found to influence pediatric patients' behavior. Those who have had negative interactions with dental care may exhibit heightened fear during dental procedures (Porritt et al., 2012). Additionally, patients who are aware of pain due to existing dental issues are more likely to display adverse behavior during their initial dental visit. Factors such as dental clinic setup and procedural approaches employed by dentists for behavior management can also impact patients' behavior during dental appointments.

Several approaches are available for managing pediatric patients' behavior in the clinic and achieving optimal dental care outcomes. One such method is the nonpharmacological approach, such as the tell‐show‐do technique (ten Berge, 2008; Porritt et al., 2012), which involves explaining the dental procedure to the pediatric patient and demonstrating the visual, auditory, and tactile aspects of the procedure. This approach is now the most employed in pediatric dental management (Anthonappa et al., 2017; Crossley & Joshi, 2002). Nonverbal communication is another approach, characterized by creating a child‐friendly and cheerful environment, engaging staff with gentle communication, and employing voice control through regulated changes in voice, volume, tone, or speed to influence and guide pediatric patients' behavior (Anthonappa et al., 2017; Greenbaum & Melamed, 1988).

By using stop signals, pediatric patients are granted some control over the clinician's actions. These signals have been shown to reduce discomfort during routine dental treatments and enhance child control (Armfield & Heaton, 2013). Some dental treatments involve complex behaviors and actions from patients, necessitating simple and concise instructions for pediatric patients. This approach is known as behavior shaping and positive reinforcement (Armfield & Heaton, 2013).

Behavioral modeling posits that individuals learn from their surrounding environment, which may positively impact their behavior. By observing the behavior of others, especially family members and older siblings, children can learn acceptable behavior in the dental clinic setting. This method employs a model, either live or filmed, to demonstrate appropriate behavior (Roberts et al., 2010). In contrast, the distraction method aims to divert a patient's focus from the dental environment or a potentially painful procedure toward another situation or task (Anthonappa et al., 2017).

Negative reinforcement through the punishment of a child could reduce dental anxiety if the stimulus perceived as aversive is removed and the appropriate behavior is immediately demonstrated. This method can reinforce a sequence of behaviors for the child in the dental clinic (Shindova, 2018). Implementing these approaches may enable dentists to modify and manage a child's behavior during dental appointments and provide optimal dental care to the child.

Given the existing knowledge gaps and the importance of managing dental anxiety and behavior in pediatric patients, this study aims to:

- Assess the behavior and anxiety of pediatric patients in the dental clinic using established behavior rating scales.
- Examine the relationship between the behavioral patterns and anxiety levels and the patient's age and gender.

By addressing these research objectives, this study will contribute valuable insights into pediatric dental anxiety and behavior management, ultimately improving dental care and outcomes for young patients.

## 2 MATERIALS AND METHODS

A cross‐sectional study was conducted at Sharjah Thumbay Dental Hospital, involving 150 pediatric patients who visited the hospital for dental treatment over a study period of 23 weeks (February 12, 2022 to July 23, 2022). Participants were selected using a consecutive sampling method, wherein every eligible patient who visited the hospital during the data collection period was included until the desired sample size was reached. The study received IRB approval (IRB/COD/STD/51/Dec‐2021) on December 23, 2021, before data collection commenced. The age range of the patients was from 2 to 14 years, and their guardians provided written consent for participation in the research. The exclusion criteria included children over 14 years old, children whose guardians refused to participate in the study, and medically compromised children.

To evaluate the pediatric patients' behavior and anxiety at the end of the dental visit, three well‐established scales were used: FBRS, Categorical rating scale, and Venham anxiety and behavioral rating scales. These scales have demonstrated good reliability and validity in previous studies and are widely used in pediatric dentistry research. The dental students who performed the evaluations received training in using these scales. After obtaining consent from the guardians, we collected demographic information (age and sex) for all participants. After each dental visit, the trained dental student evaluated the pediatric patients' behavior and anxiety using the three scales. To ensure consistency, the dental student followed a standardized protocol for conducting the evaluations.

Data analysis was performed using SPSS software (version 27). Descriptive statistics such as means and standard deviations were calculated for each scale and presented in tables and graphs. Non‐parametric tests, including Mann–Whitney *U* and Kruskal–Wallis tests, were used to compare the mean scores on the different scales according to demographic characteristics. A *p*‐value < .05 was considered statistically significant.

## 3 RESULTS

**Figure 1:**
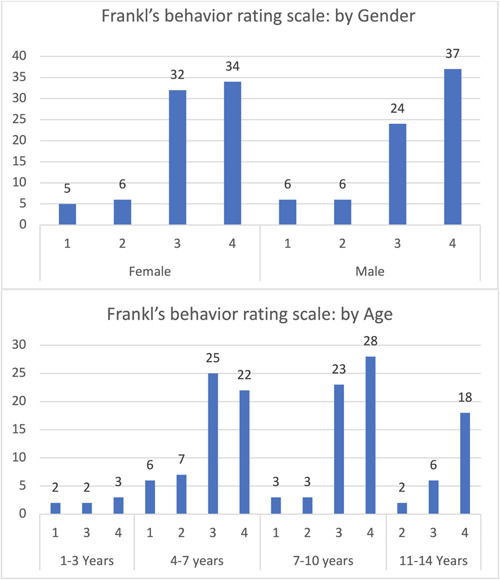
Frankl's behavior rating scale.

The majority of pediatric patients, 71 in total, were in the fourth category of FBRS, with a slightly higher prevalence among males than females. The primary age group in this category was 7–10 years, while the lowest prevalence was observed in the 1–3 years age group. The third category of FBRS ranked second, with females scoring higher than males, mainly in the 4–7 years age group. Minor differences were observed between the first and second categories of FBRS, as well as between genders in both groups (Figure 1).

**Figure 2:**
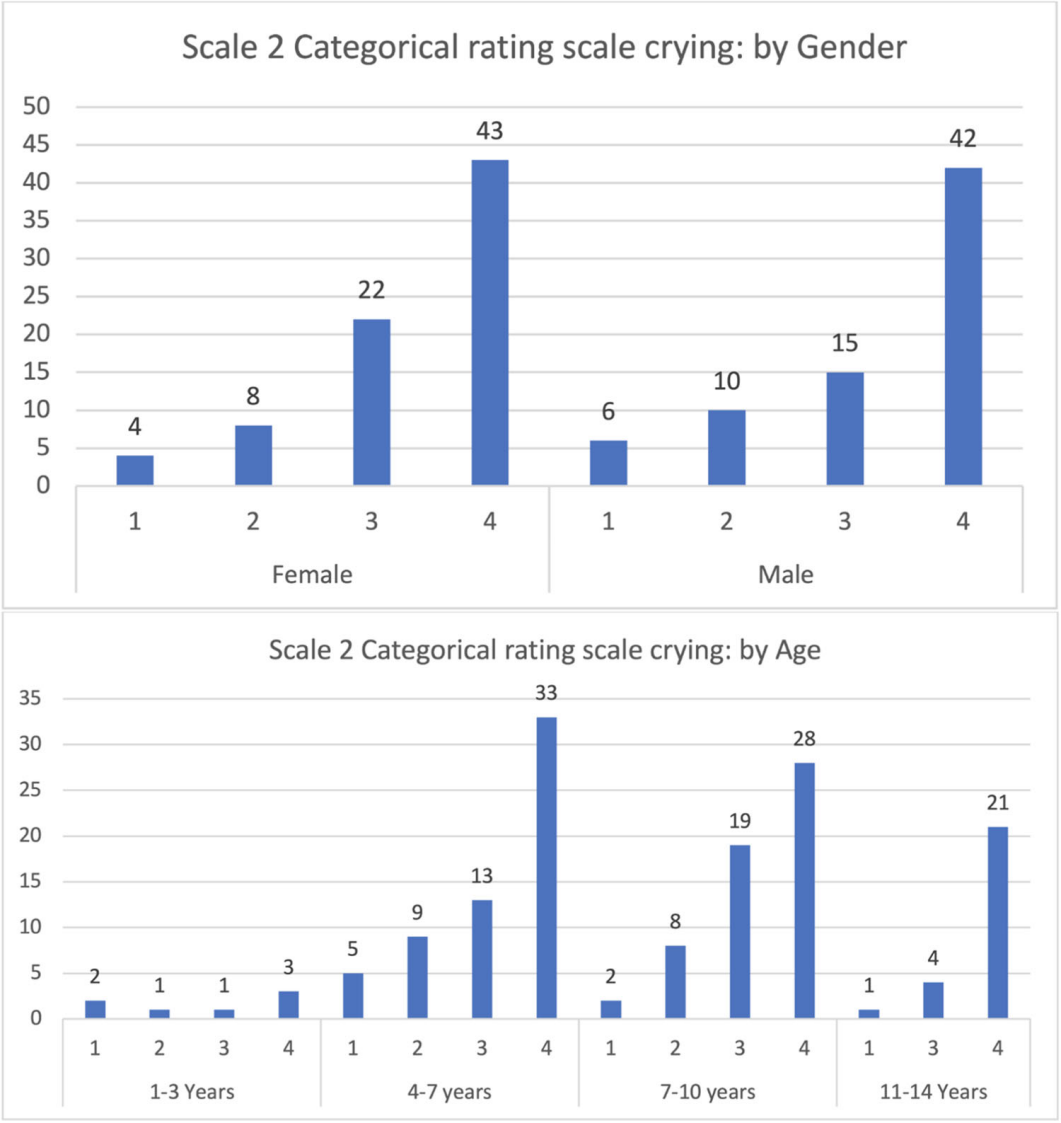
Categorical rating scale: Crying.

In the categorical rating scale for crying, most patients were in the third category (no crying), with a slight difference between genders. The 4–7 years age group showed the highest percentage, while the fourth category (screaming) had the lowest percentage, with negligible differences between genders. Males were higher than females by 1%. Children aged 1–3, 7–10, and 11–14 years had the same screaming rate, while in the 4–7 years age group, it was found to be higher (Figure 2).

**Figure 3:**
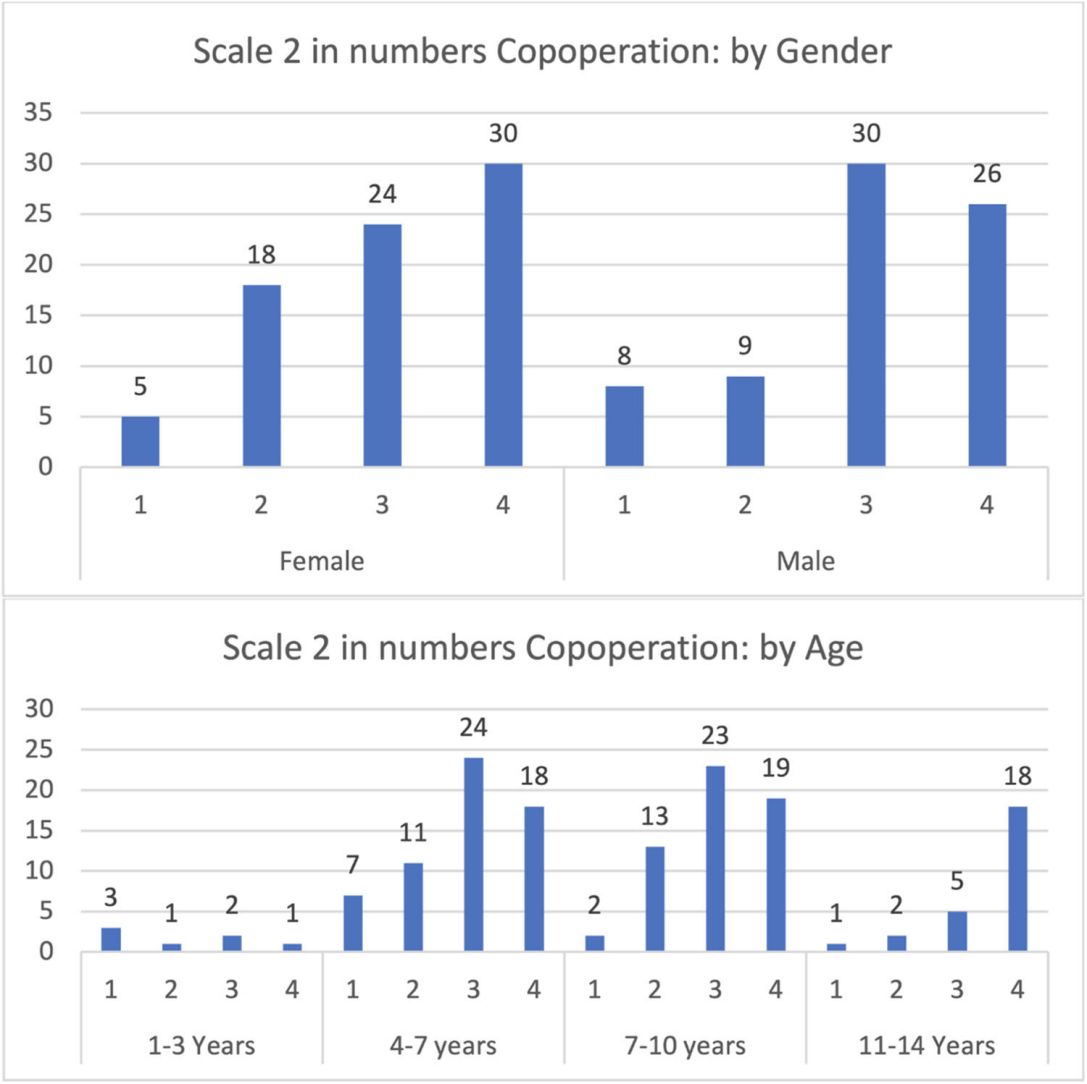
Categorical rating scale: Cooperative.

No movements were observed in both genders, with a minimal increase in females. The 7–10 years age group showed the highest percentage of no movement. Minimal differences were observed between children with no movement and those with minor or intermittent movement (Figure 3). A minority of children exhibited violent resistance or disrupted treatment, with males scoring slightly higher than females.

**Figure 4:**
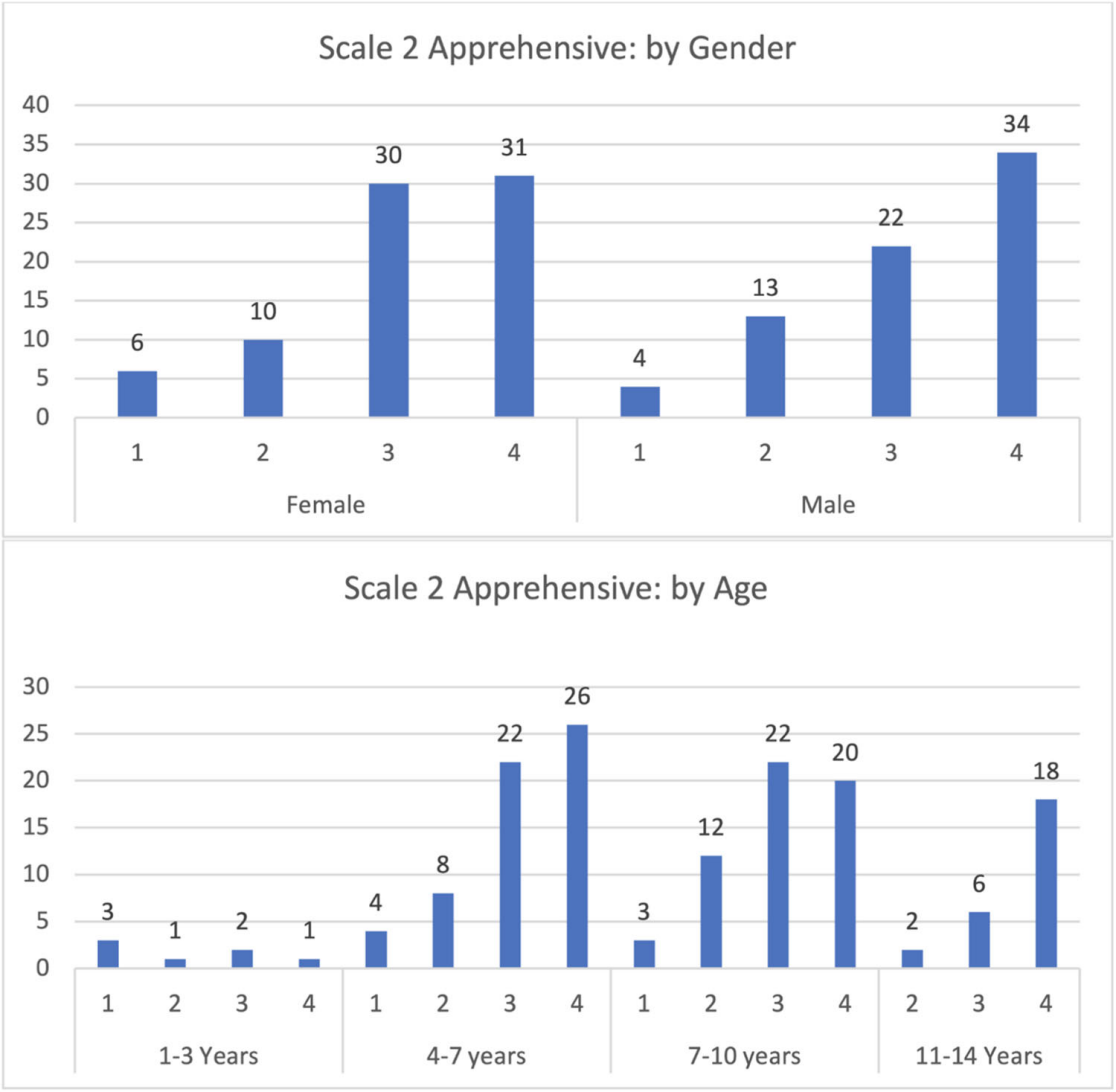
Categorical rating scale: Apprehensive.

Most children (65) were calm, relaxed, and followed instructions, with a slight difference between males and females. The majority were in the 4–7 years age group. Few children (10) were hysterical and disobeyed all instructions, with minimal differences between genders and age groups. Notably, children aged 11–14 years did not display hysterical behavior or disobey all instructions (Figure 4).

In the categorical rating scale for sleeping, the highest number were in female pediatric patients 69 of the patients were fully awake, primarily in the 4–10 years age group. Minimal differences were observed among other categories and genders for asleep/intermittent, drowsy, and sound asleep.

**Figure 5:**
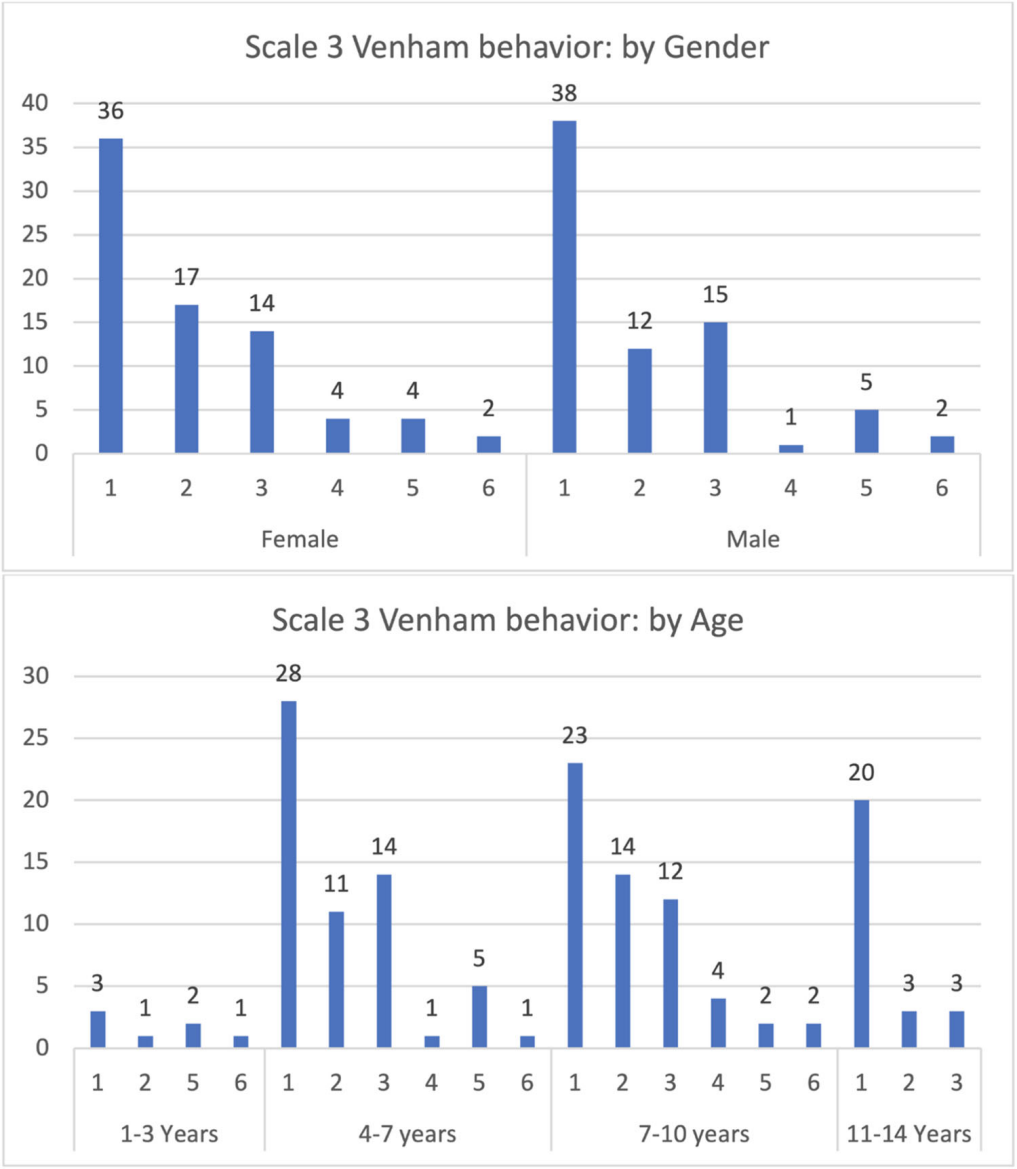
Venham: Behavior scale.

Most participants exhibited category 3 behavior in the Venham scale (total cooperation, best possible working conditions, no crying or physical protest), followed by equal percentages in categories 4 and 2, primarily in the 4–7 years age group. In the youngest children (1–3 years), categories 4 and 5 were not expressed, while in the oldest children (11–14 years), categories 1 and 6 were not expressed (Figure 5).

**Figure 6:**
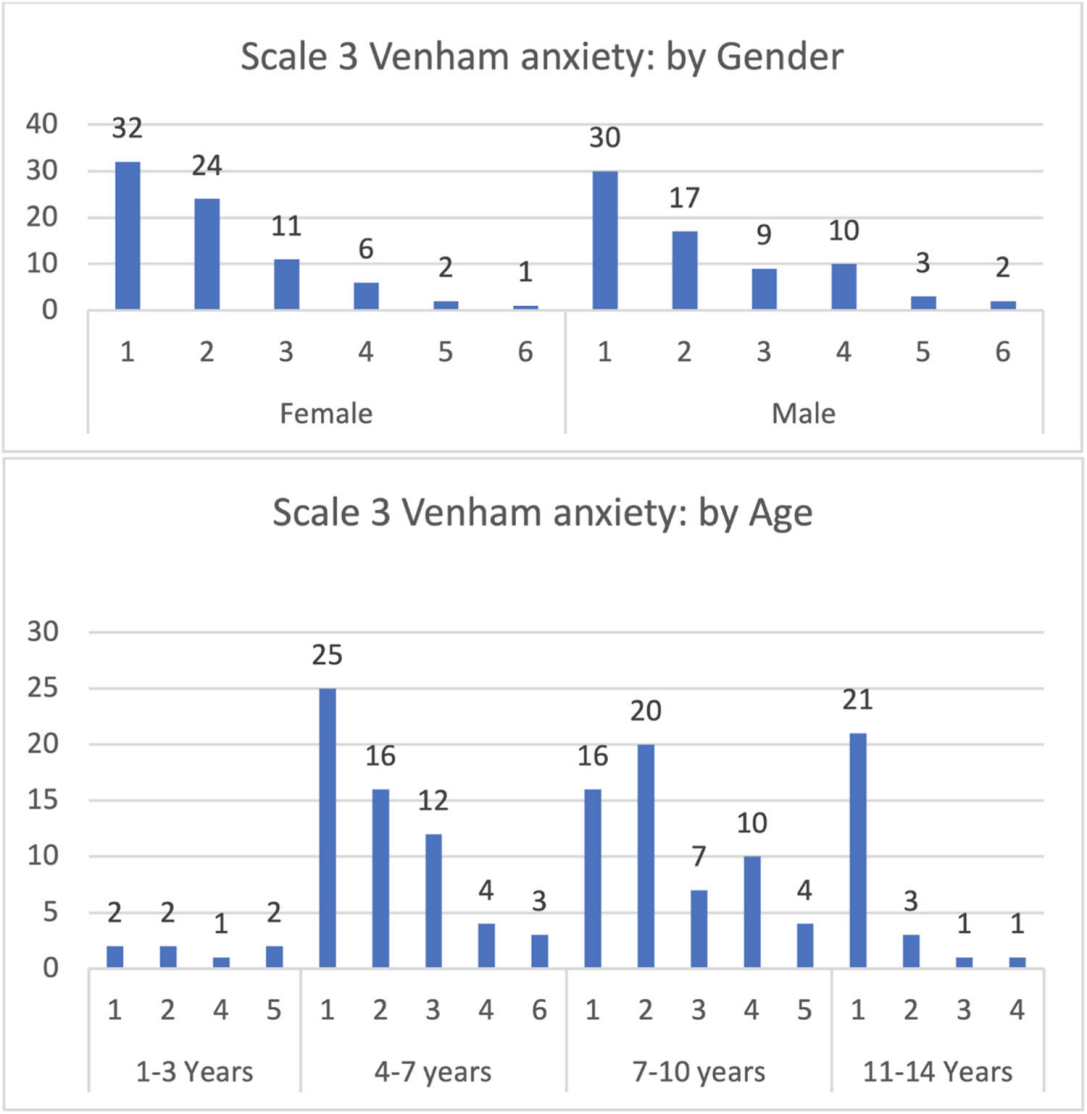
Venham: Anxiety scale.

Children exhibiting category 1 behavior (relaxed, smiling, willing, and able to converse) constituted 62 patients of the sample, primarily in the 4–7, 7–10, and 11–14 years age groups, with minimal differences among them. Category 6 displayed the lowest percentage, with children only in the 4–7 years age group. Category 5 (anxiety interferes with the ability to assess situations) was observed in children aged 1–3 and 7–10 years only. The youngest children (1–3 years) had the lowest percentage across all categories (Figure 6).

Using the Mann–Whitney *U* test, no significant differences were found between males and females in all scales utilized in the study. However, significant results were obtained in various age groups using the Kruskal–Wallis test. In Frankl's scale, the most notable outcome was observed in the oldest age group (11–14 years), with a significant difference in age (*p* = .031). In the categorical rating scale, the 11–14 years age group exhibited the most remarkable results in crying, cooperative, and apprehensive categories. All elements of this scale showed a significant relationship with age, except for sleeping: crying (*p* = .034), cooperative (*p* = .002), and apprehensive (*p* = .003). In the Venham anxiety and behavioral rating scale, significant relationships with age were found in both subscales: Venham anxiety (*p* ≤ 0.001) and behavioral (*p* = .013).

## 4 DISCUSSION

Multiple scales for measurement have been used in numerous studies on dental behavior and anxiety levels in pediatric patients (Kilinç et al., 2016; Pani et al., 2016; Riba & Al‐Zahrani, 2017; Tyagi & Sharma, 2011). The rationale for this approach stems from the inherent challenges in assessing pediatric patients' behavior and anxiety levels. Dentists typically rely on more precise measures to gauge anxiety levels in children, with such scales providing valuable insights even before treatment commencement. However, dental anxiety evaluation remains a complex endeavor due to its subjective nature, which varies among individuals. Consequently, this study utilized three distinct, internationally recognized scales (Kilinç et al., 2016).

Sivakumar and Gurunathan (Sivakumar, 2019) incorporated FBRS in their research, revealing a positive correlation between age and favorable outcomes in both behavior and anxiety within the clinical setting, consistent with our findings. In contrast, the results of a 2016 study by Kilinç and Akay were discordant with our observations (Kilinç et al., 2016).

Regarding the gender distribution of FBRS, our outcomes aligned with those of Sivakumar and Gurunathan. Their investigation demonstrated that females scored higher on the third element of Frankl's scale compared to males, whereas males surpassed females in the fourth element, possibly attributable to increased sensitivity in females (Sivakumar, 2019). Additionally, our study revealed a maximum 5% discrepancy between genders in the third element of Frankl's scale, with no significant differences observed across other age groups. These findings concur with those of a 2016 study by Kilinç et al. (2016).

The implementation of Venham behavior and anxiety scales effectively documented pediatric patients' behavior and anxiety, offering a subjective evaluation to inform the clinician. Interestingly, in the 4–7 years age group, the scales indicated that more prominent protest behaviors (e.g., crying and hand signals) exceeded milder manifestations (e.g., soft verbal protest or quiet crying as discomfort signals). The inverse relationship was observed in the 7–10 years age group, with both categories diminishing in the 11–14 years age group. This trend can be attributed to the positive correlation between education level and improved behavior, as well as reduced anxiety among children. As knowledge accrues with age, better behavior and diminished anxiety during dental treatment are anticipated (Pani et al., 2016; Riba & Al‐Zahrani, 2017; Sahithi et al., 2021). These patterns elucidate the significant results obtained from most behavior and anxiety scales concerning age, as determined by the Kruskal–Wallis test.

Crying, a variable within the categorical rating scale in our study, exhibited a minor gender‐based difference. The 4–7 year age group displayed increased mild or intermittent crying, potentially due to heightened danger recognition and demonstration attempts by children, despite their limited expressive capacity. In terms of cooperation, males exhibited greater resistance and treatment disruption than females, possibly reflecting a natural inclination for situational control. The majority of children in our study were classified as calm, relaxed, and compliant with instructions, which may be attributed to the judicious selection of management strategies to circumvent traumatic dental experiences. The preponderance of fully awake patients can be explained by the scheduling of mid‐morning weekend appointments when children typically arrive at the clinic refreshed and amenable to treatment (Riba & Al‐Zahrani, 2017).

Dedicated time and effort are essential to maintaining pediatric patients' calm and reassurance within the dental clinic. Before treatment, awareness of a child's behavior and anxiety levels enables dentists to anticipate and plan for behavior and anxiety‐related responses, empowering them to implement control measures if necessary (Kilinç et al., 2016).

## 5 CONCLUSION

The pediatric patients who took part in this study exhibited heightened anxiety when attending dental clinics; this study emphasizes the significant anxiety experienced by pediatric patients in dental settings. It underscores the importance of understanding child behavior and utilizing effective communication with both children and their guardians. Dental professionals should consider implementing strategies such as behavioral modeling and nonpharmacological techniques to manage child behavior during visits. By doing so, they can improve the quality of dental care provided to pediatric patients. Further research is required to develop more effective strategies tailored to different pediatric populations, aiming to enhance dental care outcomes for our young patients.

## AUTHOR CONTRIBUTIONS

**Rana A. Al Homoud**: Proposal writing; materials and methods; statistical analysis; results writing; discussion; finalizing the research content; review and editing. **Amani K. Alshellatie**: Proposal writing; background; conclusion; reviewing and revising the research content. **Abdullah S. Alzumaie**: Data collection; abstract writing; reviewing the final manuscript. **Sura A. Al Bayati**: Writing; reviewing and editing; project supervision.

## CONFLICT OF INTEREST STATEMENT

The authors declare no conflict of interest.

## Data Availability

Materials form all data of participant from patient in Thumbay Sharjah dental hospital, by the observations of the patient. Data are available on request from the authors.
